# High fat diet induces airway hyperresponsiveness in mice

**DOI:** 10.1038/s41598-018-24759-4

**Published:** 2018-04-23

**Authors:** Kathrin Fricke, Marcela Vieira, Haris Younas, Mi-Kyung Shin, Shannon Bevans-Fonti, Slava Berger, Rachel Lee, Franco R. D’Alessio, Qiong Zhong, Andrew Nelson, Jeff Loube, Ian Sanchez, Nadia N. Hansel, Wayne Mitzner, Vsevolod Y. Polotsky

**Affiliations:** 1 Division of Pulmonary and Critical Care Medicine, Department of Medicine, Johns Hopkins University School of Medicine, Baltimore, MD USA; 2 Department of Environmental Health and Engineering, Johns Hopkins Bloomberg School of Public Health, Baltimore, MD USA; 3 Division of Pulmonary Medicine, Department of Internal Medicine, Hannover Medical School, Hannover, Germany

## Abstract

The experiment was conducted to examine the effect of a high fat diet (HFD) on airway hyperresponsiveness (AHR) in mice. Twenty-three adult male C57BL/6 J mice were fed with HFD or regular chow diet for two weeks. The total respiratory resistance was measured by forced oscillation technique at baseline and after methacholine aerosol challenge at 1, 3, 10 and 30 mg/mL. Bronchoalveolar lavage (BAL) was performed. Lipid levels and lipid peroxidation in lung tissue were measured along with gene expression of multiple cytokines. Lungs were digested, and IL-1β secretion by pulmonary macrophages was determined. HFD feeding resulted in 11% higher body weight compared to chow. HFD did not affect respiratory resistance at baseline, but significantly augmented airway responses to methacholine compared to chow diet (40.5 ± 17.7% increase at 30 mg/ml methacholine, p < 0.05). HFD induced a 3.2 ± 0.6 fold increase in IL-1β gene expression (p < 0.001) and a 38 fold increase in IL-1β secretion in the lungs. There was no change in BAL and no change in any other cytokines, lipid levels or lipid peroxidation. Hence, HFD induced AHR in mice prior to the development of significant obesity which was associated with up-regulation of pulmonary IL-1β.

## Introduction

Asthma is one of the most common diseases and the prevalence of asthma continues to increase, which has been attributed to the epidemics of obesity^1–3^. Asthma in obesity appears to be different from typical TH2 driven allergic asthma demonstrating a poor response to inhaled corticosteroids^4^. Possible mechanisms include breathing at lower lung volumes, altered airway structure, increased airway oxidative stress, and greater systemic inflammation^5^. Up-regulation of the NLRP3 inflammasome and IL-1β has been implicated in asthma in high fat diet (HFD) induced obesity^6^. HFD is pro-inflammatory due to direct effects of free fatty acids^7^. However, the effect of high fat diet per se on airway hyperresponsiveness (AHR) has not been investigated. We hypothesize that high fat diet induces inflammation which can affect AHR independent of obesity.

## Methods

### Experimental animals

Twenty-three adult male C57BL/6 J mice, 10 weeks of age (Jackson Laboratory, Bar Harbor, MA) were fed with HFD (TD 03584, Teklad WI, 5.4 kcal/g, 35.2% fat, 58.4% kcal from fat, n = 10) or chow diet (3.0 kcal/g, 4.4% fat, 13% kcal from fat, n = 13) for 14 days. Details on HFD composition are provided in Supplemental Table 1. HFD was refrigerated at 4–8 °C before it was added to the cages. Food and water was provided *ad libitum*. Mice were housed in a standard laboratory environment at 22 °C in the 12 h light/dark cycle (9 am–9 pm lights on/9 pm–9 am lights off). In order to assure reproducibility of the measurements, mice were separated in two batches (Batch 1, HFD, n = 5, chow diet, n = 6; Batch 2, HFD, n = 5, chow diet, n = 7), which were studied six months apart using different batches of HFD. The study was approved by the Johns Hopkins University Animal Use and Care Committee (Protocol # MO15M257) and complied with the American Physiological Society Guidelines for Animal Studies.

### Physiological measurements and Histology

On day 14 mice were anesthetized with ketamine/xylazine i.p., tracheostomized and the total respiratory resistance (Rrs) was measured by forced oscillation technique (Flexivent) at baseline and after methacholine aerosol challenge at 1, 3, 10 and 30 mg/mL as described^8,9^. Blood was collected from the aorta, bronchoalveolar lavage (BAL) was performed with 2 × 0.8 mL of sterile phosphate-buffered saline (PBS) through a tracheal cannula. The thorax was opened, and the right lung was tied off, dissected free and immediately frozen in liquid nitrogen and stored at −80 °C. The remaining left lung was inflated with formalin at 26 cmH2O pressure for 20 min, tied off and placed inflated in formalin for 2 days. Left lung volumes were measured by water replacement.

For histology the left lung was dehydrated in ethanol and embedded in paraffin. For morphometry, 5-μm-thick sections were cut from transverse blocks and stained with Masson trichrome.

### Blood, Plasma and Lung Tissue Analysis

Complete blood counts (CBC) were determined. Triglycerides and free fatty acids (FFA) were measured in lung homogenates and plasma with kits from Wako Inc (Richmond, VA). Plasma insulin and leptin were measured with kits from Alpco Diagnostics (Salem, NH) and Abcam (Cambridge, MA), respectively. Blood glucose levels were measured with a glucometer (ACCU-CHECK Aviva Plus, Roche, Indianapolis, IN). Total RNA was extracted from lung tissue with a Trizol reagent (Life Technologies, Rockville, MD). cDNA was produced from total RNA using Advantage RT for PCR kit from Clontech (Palo Alto, CA). Real time PCR was performed for the cytokine panel, including interleukins (IL) 1β, 4, 5, 6, 10, 13, 17, TNF-α, IL-21, IL-23, adiponectin, leptin, fork head box protein P3 (FOXP3), matrix metallopeptidase (MMP 9), as well as toll-like receptors (TLR)−2 and 4 with premade primers from Invitrogen (Carlsbad, CA), and Taqman probes from Applied Biosystems (Foster City, CA) using 18 S as a housekeeping gene (Supplemental Table 2).

Custom made 18 S primers were forward 5′-CTCTTTCGAGGCCCTGTAATTGT-3′, reverse, 5′-AACTGCAGCAACTTTAATATACGCTATT-3′ and the probe 6FAM-AGTCCACTTTAAATCCTT. Target mRNA level was normalized to 18 s rRNA, using the formula: Target/18 s = 2^Ct(18s)–Ct(target)^. Activity of nuclear factor κB (NF- κB) was derived from the of phosphorylated to total IκBα protein with a kit from Abcam. Lipid peroxidation in lungs was measured by malondialdehyde level with a kit from Abcam.

### Cytokine Secretion and Flow Cytometry

In a subset of mice left lungs were harvested, minced and placed in gentle MACS Dissociator (Miltenyi Biotec), and digested using Collagenase type 1 (Worthington) and DNase I (Sigma Aldrich, St. Louis, MO) for 10 minutes at 37 °C. The lung digests were passed through a 70-μm nylon cell strainer (Becton Dickinson, Franklin Lakes, NJ), and erythrocytes were subsequently lysed using RBC Lysis Buffer (eBioscience, San Diego, CA). The cells were counted and cells viability was assessed by Trypan Blue staining. Then, 2 × 10^6^ of viable cells were seeded in 96 well plates in the presence of DMEM + 10% FBS + Pen/Strep 1:100 medium. Two hours later, non-adherent cells were removed and 100 ul of the medium were added to the attached cells. The cells were incubated for 24 hours at 37 °C, the media was collected, centrifuged to remove cells and debris, and IL-1β, TNF-α and IL-6 secretion was measured with an ELISA kit (R&D systems). For flow cytometry, cells were washed with FACS buffer (PBS + 0.5% BSA) and incubated with PE-Cy CD64 Ab. Then, non-specific staining of Fcγ III/II receptors was blocked with Fc Block-2.4G2 (BD Biosciences — Pharmingen) Ab. The following Abs (BD Biosciences — Pharmingen) were used for cell phenotyping: PerCp Cy 5.5-conjugated anti-CD11c, PE-CF594-conjugated anti-CD11b, APC-Cy7-conjugated anti-MHCII, BV421-conjugated anti-SigF, BV605-conjugated anti-Ly6c, BV510-conjugated anti-Ly6g, BV395-conjugated anti-CD4 and BV737-conjugated anti-8 and respective isotype Abs. Lymphocytes, monocytes, neutrophils, alveolar and interstitial macrophages were gated with characteristic low forward scatter/side scatter, using a FACSAria instrument and FACSDiva for data acquisition (Becton Dickinson) and Flowjo for analysis (Tree Star Inc.) as previously described^10^.

### Data Availability

All data generated or analyzed during this study are included in this published article.

### Statistical analysis

All values are reported as means ± SEM. All the data in the study were checked for normality with a chi-square goodness of fit test. Statistical comparisons on non-normally distributed values were performed by the Mann-Whitney U test. Statistical significance for normally distributed values was determined by student’s t-test or two-way analysis of variance test (ANOVA) with the Bonferroni correction when appropriate. A p-value of < 0.05 was considered significant. Statistical analysis was performed using PRISM 7 and Stata.

## Results

Baseline characteristics of the experimental animals are described in Table 1. HFD feeding for 2 weeks resulted in 11% higher body weight than in chow-fed mice doubling the size of epididymal and retroperitoneal fat pads. Of note, inguinal fat pads were not increased. HFD feeding induced hyperglycemia and increases in plasma insulin and leptin levels, whereas plasma triglyceride were unchanged (Table 1). There was no difference in CBC (Supplemental Table 3). Diet did not affect left lung volumes which were 0.16 ml ± 0.03 on a chow diet and 0.18 ± 0.03 on HFD.

**Table 1:** Basic characteristics, and plasma metabolic parameters in regular chow and high fat diet mice.

		Chow diet (n = 13)	High Fat Diet (n = 10)
Initial Body weight (g)		26.09 ± 0.74	25.67 ± 0.74
Final Body weight (g)		26.65 ± 0.59	29.21 ± 0.77*^†^
Daily food intake (KJ/mouse)		47.9 ± 12.42	70.9 ± 6.32
Glucose levels (mg/dL)		140.1 ± 4.15	188.8 ± 8.48***
Plasma triglycerides (mg/dL)		119.79 ± 34.17	123.04 ± 71.61
Plasma free fatty acids (mmol/L)		0.44 ± 0.02	0.51 ± 0.03
Plasma insulin (ng/mL)		0.111 ± 0.007	0.167 ± 0.029*
Plasma leptin (ng/mL)		5.6 ± 0.5	18.2 ± 1.8**
Epidydimal fat pads weight (g)	Right	0.223 ± 0.05	0.481 ± 0.16***
	Left	0.213 ± 0.04	0.455 ± 0.167***
Retroperitoneal fat pads weight(g)	Right	0.054 ± 0.01	0.093 ± 0.03**
	Left	0.051 ± 0.015	0.127 ± 0.064**
Inguinal fat pads weight (g)	Right	0.117 ± 0.011	0.115 ± 0.044
	Left	0.123 ± 0.023	0.109 ± 0.04

*,**, and ***denote p < 0.05, <0.01 and <0.001, respectively, compared to chow diet; ^†^denotes p < 0.05 for the weight gain during the experiment in the high fat diet group.

**Figure 1:**
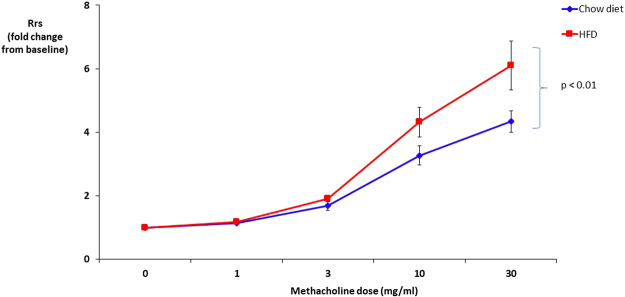
High fat diet (HFD) increased total resistance of the respiratory system **(**Rrs) in response to methacholine. The Rrs values were normalized to baseline (no significant difference between groups at baseline).

Pulmonary function testing revealed no difference in total lung resistance at baseline between mice fed chow and HFD (Rrs of 0.69 ± 0.04 cm H_2_O*s/ml and 0.63 ± 0.03 cm H_2_O*s/ml, respectively). HFD significantly augmented airway responses to methacholine compared to chow diet (Fig. 1).

**Figure 2:**
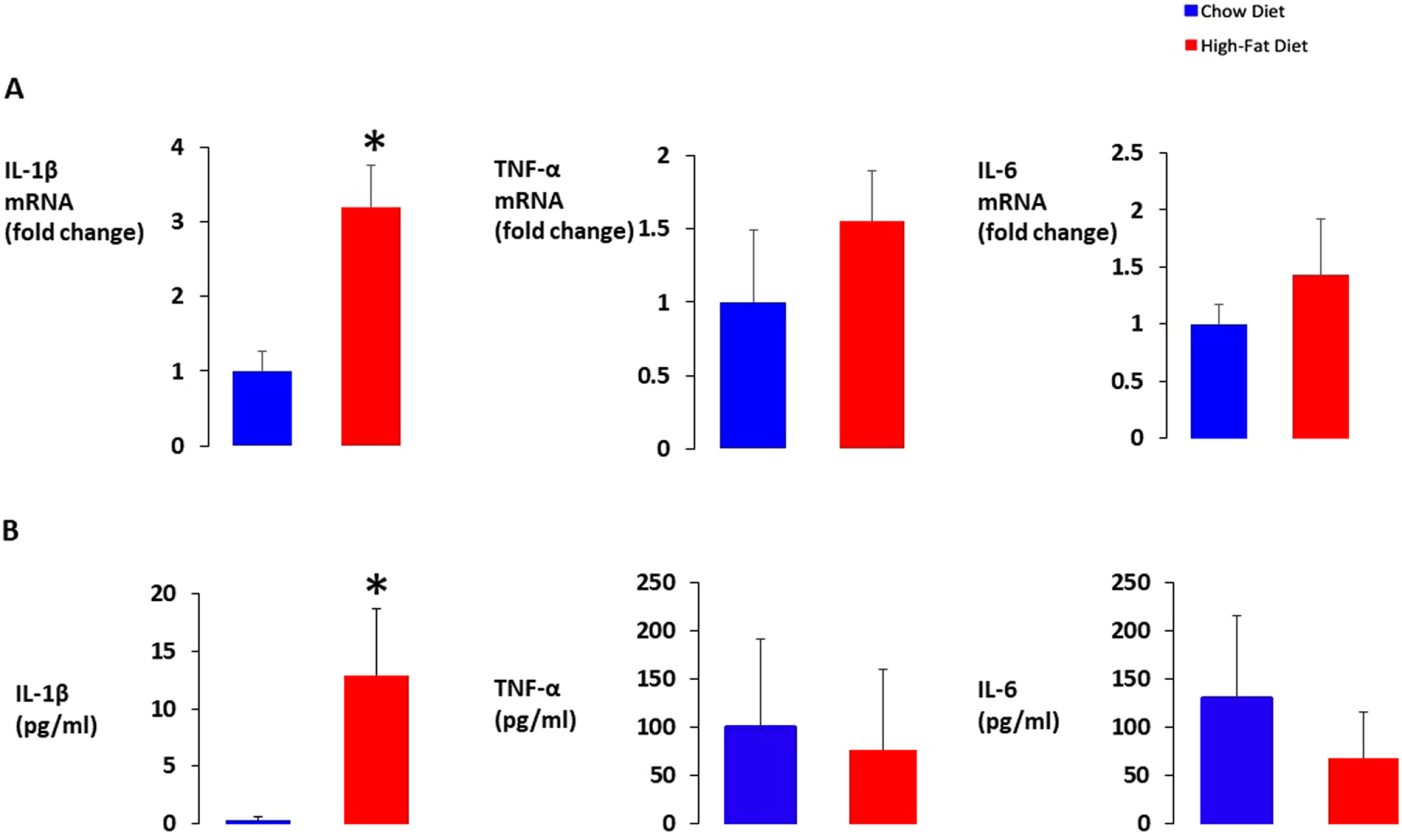
The effect of high fat diet (HFD) feeding for 14 days on **(A)** interleukin 1β (IL-1β), tumor necrosis factor α (TNF-α) and interleukin 6 (IL-6) mRNA levels in lung tissue; **(B)** IL-1β, TNF-α and IL-6 protein secretion to the media by adherent cells isolated from the lung single cell suspension. *p < 0.05 for the difference with a regular chow diet.

**Figure 3:**
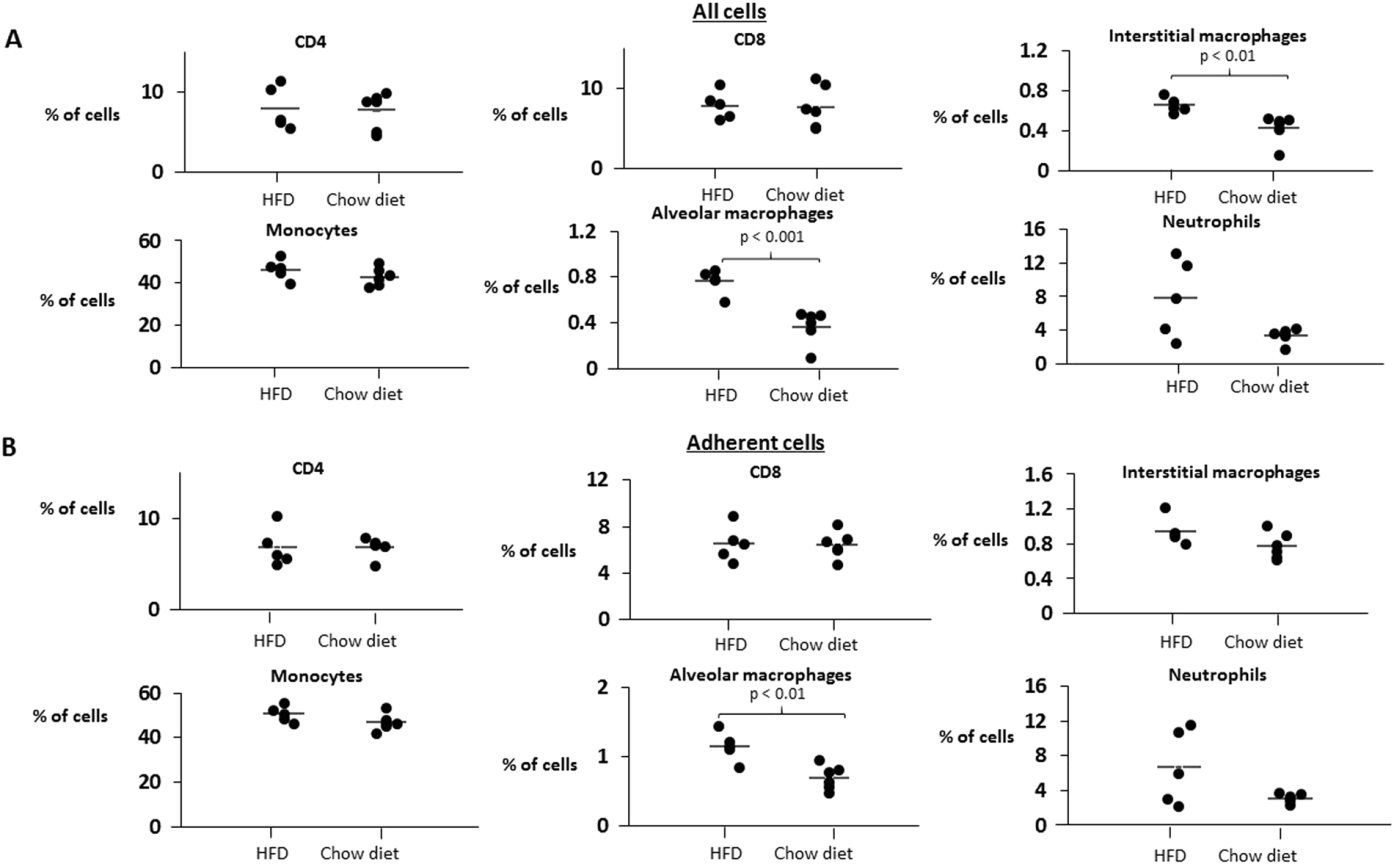
The effect of high fat diet (HFD) feeding on the leukocyte population in the mouse lungs. CD4, CD8 lymphocytes, interstitial macrophages, monocytes, and alveolar macrophages were identified by flow cytometry according to protocol described by Misharin *et al*. (ref.^10^) in **(A)** total lung leukocyte suspension and **(B)** the adherent cell population as described in methods.

Compared to chow diet, HFD did not affect lung lipid content, lipid peroxidation (malondialdehyde), BAL cell count and differential, TNF-α and IL-6 lung mRNA expression and secreted protein levels **(**Fig. 2**)** and adiponectin mRNA levels **(**Table 2). Histological examination show no evidence of inflammation or fibrosis in HFD mice (Supplemental Fig. 1). Mice on both diets showed no detectable pulmonary expression of pro-inflammatory or pro-allergic IL-4, IL-5, IL-13, IL-17, IL-21, IL-23, leptin, MMP 9, TLR 2, TLR 4 and anti-inflammatory IL-10 and FOXP3. There was no indication of upregulation of NF-κB by HFD, because the IκB subunit showed similar levels of phosphorylation in both dietary groups. In contrast, HFD induced a significant 3.2 ± 0.6 fold increase in total lung IL-1β mRNA compared to chow diet (p = 0.001), and this difference remained significant after the Bonferroni correction for multiple comparisons **(**Fig. 2A**)**. Most remarkably, HFD induced a 38-fold increase in IL-1β secretion by adherent cells isolated from the lungs, presumably monocytes and macrophages **(**Fig. 2B**)**. The flow cytometry of total lung digest showed that HFD induced a 1.3-fold increase in the percentage of interstitial macrophages and 1.7-fold increase in the percentage of alveolar macrophages in lung tissue **(**Fig. 3A**)**. Moreover, HFD mice had a 1.4-fold increase in the percentage of adherent alveolar macrophages compared to mice on a chow diet **(**Fig. 3B**)**.

**Table 2:** Lung metabolic parameters and bronchoalveolar lavage (BAL) data in regular chow and high fat diet mice.

		Chow diet (n = 13)	High Fat Diet (n = 10)
Triglycerides in lung tissue (µg/mg)		12.96 ± 1.08	14.55 ± 2.20
Free fatty acids in lung tissue (µmol/mg)		0.039 ± 0.003	0.038 ± 0.001
Lung malondialdehyde (uM/mg)		36.08 ± 3.83	30.73 ± 4.28
Phosphorylation of IκBα (% of total)		2.7 ± 0.9	2.6 ± 0.9
Lung adiponectin mRNA (fold change)		1.00 ± 0.19	0.63 ± 0.18
BAL cell count (cells/mL)		36212 ± 6775	31666 ± 2317
BAL differential (% of total)	Epithelial cells	10.08 ± 1.39	12.58 ± 1.92
	Macrophages	89.28 ± 1.43	86.88 ± 1.91
	Eosinophils	0	0
	Basophils	0	0
	Neutrophils	0.05 ± 0.02	0.02 ± 0.02
	Lymphocytes	0.59 ± 0.14	0.52 ± 0.2

## Discussion

To our knowledge this is the first study demonstrating that high fat diet induces airway hyperresponsiveness (AHR) early in the time course, only after two weeks of feeding. Another novel finding of the study is that HFD feeding induces IL-1β expression in lung tissue and increases IL-1β secretion in the lungs prior to the development of significant obesity.

Numerous factors may account for increased AHR in obesity. Several phenotypes of obesity associated asthma have been identified, including obesity-induced structural changes in lung volumes and airways; pre-existing allergic asthma complicated by obesity; neutrophilic asthma and asthma induced by obesity-related environmental factors (e.g. high fat diet)^11^. The latter phenotype has been associated with increased IL-1β inflammatory response^11^. However, it has been previously unknown whether HFD can lead to AHR early in time course. Our data in a non-allergy prone strain of mice showed that HFD activates the IL-1β pathway specifically in the lungs, prior to the development of significant obesity. Previous data showed that IL-1β knockout or receptor blockade abolished an increase of AHR in mice with severe diet-induced obesity^6^. Furthermore, IL-1β may directly potentiate cholinergic bronchoconstriction^12^. There was no signs of eosinophilic or neutrophilic inflammation in BAL associated with obese asthma^13,14^ and there was no increase in TH2 cytokines like IL-4. We observed an increase in the percentage of pulmonary macrophages in mice fed with a HFD. However, this increase was small compared to a striking increase in IL-1 β levels. Thus, our data suggest that up-regulation of IL-1β in the lungs is implicated in causality of HFD-induced asthma.

What would be a potential mechanism by which dietary fat induces IL-1β in the lungs? In patients with asthma, a high fat diet meal increases airway inflammation and decreases the airway response to bronchodilators^15^. The pulmonary microvascular endothelial cells are responsible for triglyceride uptake and triglyceride clearance^16^. FFA may activate the NF-κB pathway followed by IL-1β up-regulation^17^. Another possible mechanism would be a high fat diet induced activation of the NLRP3 inflammasome through fatty acids or cholesterol crystals in pulmonary macrophages resulting in IL-1β production. However, we did not find an increase in FFA or activation of the NF- κB pathway. Also, the lack of an effect of HFD on IL-17, an indispensable component of the inflammasome may argues against this mechanism^6^.

Up-regulation of IL-1β is not the only possible mechanism by HFD-induced bronchial reactivity. HFD induced a 3-fold increase in plasma leptin. In patients with asthma, high leptin have been independently associated with the disease severity^18^. In a mouse model leptin treatment induced the TH2 response and allergic inflammation of the airways^18^. Of note, leptin may also increase IL-1β^19,20^, which would be consistent with current findings. Finally, our data suggest that HFD feeding for 2 weeks induced early manifestations of the metabolic syndrome including visceral fat deposition with an increased size of visceral fat pads (epidydimal and retroperitoneal), but not subcutaneous fat pads (inguinal), hyperglycemia and hyperinsulinemia **(**Table 1**)**. Coexistence of metabolic syndrome and asthma is well documented in the literature^21^. Low grade systemic inflammation observed in the metabolic syndrome may contribute to the development of obese asthma via IL-1β and other pathways. Furthermore, treatment of insulin resistance, a hallmark manifestation of the metabolic syndrome, may have a beneficial effect in obese asthma^22^.

### Limitations

The main limitation of our study is that the role of IL-1β in HFD-induced asthma is not confirmed in mechanistic experiments using IL-1β knock out mice or IL-1β receptor blockers. However, this causal relationship has been shown previously by other investigators in mice with diet-induced obesity^6^. Future experiments using IL-1β receptor blockers are needed.

### Conclusion and implications

Our data suggests that HFD may rapidly induce airway hyperresponsiveness prior to the development of significant obesity with early involvement of IL-1β. Given that IL-1β production is not responsive to steroids in severe asthma^23,24^, our data imply that dietary fat restriction may be an important adjunct to other therapies used in obese asthma.

## Electronic supplementary material

Supplementary Information
