# Supplementary material for: High fat diet induces airway hyperresponsiveness in mice
Source: Sci Rep. 2018 Apr 23;8:6404. doi: 10.1038/s41598-018-24759-4 (PMC5913253; doi:10.1038/s41598-018-24759-4)
Supplement: Supplementary file 1 — Supplementary Information [file 41598_2018_24759_MOESM1_ESM.pdf]

High fat diet induces airway hyperresponsiveness in mice

Kathrin Fricke[1,3*], Marcela Vieira[1*], Haris Younas[1*], Mi-Kyung Shin[1], Shannon Bevans-Fonti[1], Slava Berger[1], Rachel Lee [1], Franco R D'Alessio [1], Qiong Zhong[1], Andrew Nelson[2], Jeff Loube[2], Ian Sanchez[2], Nadia N Hansel[1], Wayne Mitzner[2], and Vsevolod Y Polotsky[1**]

1: Division of Pulmonary and Critical Care Medicine, Department of Medicine, Johns Hopkins University School of Medicine, Baltimore, MD;
2: Department of Environmental Health and Engineering, Johns Hopkins Bloomberg School of Public Health, Baltimore, MD;
3: Division of Pulmonary Medicine, Department of Internal Medicine, Hannover Medical School, Germany

*These authors contributed equally to the manuscript
** Corresponding author, vpolots1@jhmi.edu

Supplemental Figure 1. Representative images of the lungs of C57BL/6J mice on a regular chow diet (A, B) and a high fat diet (C, D). Masson Trichrome. Original magnification 2.5X (A, C) and 10X (B, D). Framed areas at low magnification (A and C) are shown at high magnification (B and D respectively). No significant inflammation is seen. Collagen staining in blue surrounds the pulmonary arteries and the airways in both dietary groups.

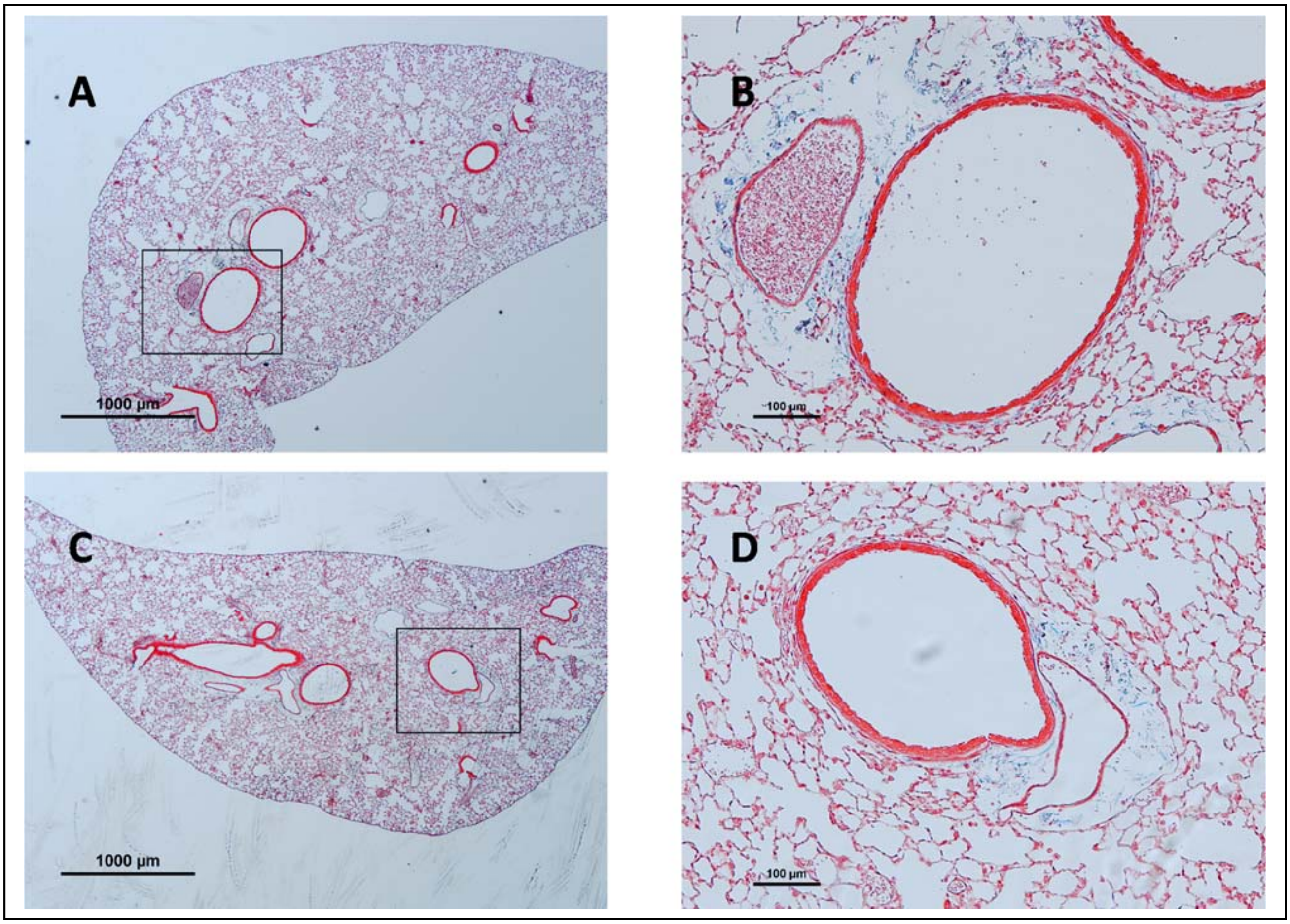

Supplemental Table 1. Fat composition in High Fat Diet

FAT COMPOSITION		PERCENTAGE (%)
**SATURATED FATTY ACIDS**		**38**
	Palmitic acid	23
	Stearic acid	13.3
	Myristic acid	1.32
	Margaric acid	0.38
**MONOUNSATURATED FATTY ACIDS**		**41.6**
	Oleic acid	36.6
	Eicosenoic acid	0.62
	Palmitoleic acid	1.94
	Vaccenic acid	2.42
**POLYUNSATURATED FATTY ACIDS**		**18**
	Linoleic acid	16.8
	Eicosadienoic acid	0.65
	Arachidonic acid	0.23
**OTHER FATTY ACIDS**		**2.4**

Supplemental Table 2. Primers and probes real time reverse transcriptase PCR

Gene	Gene Bank number	Assay ID
TNF-α	NM_001278601.1	Mm00443258_m1
Leptin	NM_008493.3	Mm00434759_m1
MMP9	NM_013599.3	Mm00442991_m1
TLR2	NM_011905.3	Mm00442346_m1
TLR4	NM_021297.2	Mm00445273_m1
IL1- β	NM_008361.3	Mm00434228_m1
IL-4	NM_021283.2	Mm00445259_m1
IL-5	NM_010558.1	Mm00439646_m1
IL-6	NM_031168.1	Mm00446190_m1
IL-10	NM_010548.2	Mm01288386_m1
IL-13	NM_008355.3	Mm00434204_m1
IL17a	NM_010552.3	Mm00439618_m1
IL-21	NM_001291041.1	Mm00517640_m1
IL-23a	NM_031252.2	Mm00518984_m1
18s	X00686	Custom made (see Methods)

Supplemental Table 3. Complete blood count in chow and high fat diet mice

	**Chow diet (n = 5)**	**High fat diet (n = 5)**
RBC (M/μL)	10.59 ± 0.48	11.158 ± 0.66
HgB (g/dl)	15.7 ± 0.578	16.3 ± 0.966
MCV (fL)	52.4 ± 0.48	52.7 ± 0.393
Platelets (k/μL)	703.8 ± 475.5	1053 ± 93.7
WBC (k/μL)	3.206 ± 1.08	3.622 ± 0.46
Neutrophils (k/μL)	0.344 ± .107	0.32 ± 0.136
Lymphocytes (k/μL)	2.416 ± 0.95	3.104 ± 0.425
Monocytes (k/μL)	0.3 ± 0.14	0.134 ± 0.115
Eosinophils (k/μL)	0.146 ± 0.133	0.064 ± 0.04
Basophils (k/μL)	0	0
